# Safety of Augmentation Mastopexy in a Teaching Environment: Complication Analysis

**DOI:** 10.1093/asjof/ojag090

**Published:** 2026-05-20

**Authors:** Francesco Marena, Aline Carrer Bortolini, Alcemar Maia Souto, Farid Hakme, Tito Brambullo

## Abstract

**Background:**

Breast hypoplasia and ptosis, whether congenital or acquired, may significantly affect body image and quality of life. Augmentation mastopexy is an effective corrective procedure but is technically demanding and associated with a recognized risk of complications, raising concerns regarding its role in surgical training.

**Objectives:**

The aim of this study was to evaluate the incidence and type of complications following augmentation mastopexy performed by plastic surgery residents under direct attending supervision and to assess the implications for training within an academic setting.

**Methods:**

A retrospective analysis was conducted of 289 consecutive augmentation mastopexy procedures performed between January 2020 and January 2024, with a minimum follow-up of 12 months. All procedures were performed by residents under the continuous supervision of experienced plastic surgeons. Data regarding patient characteristics, surgical technique, implant selection, and postoperative complications were collected and analyzed.

**Results:**

Forty-six complications were recorded, corresponding to an overall complication rate of 15.9%. Minor complications included suture dehiscence (4.84%), secondary ptosis (3.4%), asymmetry (3.11%), hematoma (1.3%), and seroma (1.3%). Major complications included capsular contracture (0.7%) and infection (1.0%). Reoperations were primarily required for major complications or patient-reported aesthetic dissatisfaction. Overall outcomes were comparable to those reported in the literature.

**Conclusions:**

Within a structured academic program with direct attending supervision, augmentation mastopexy can be safely performed by residents, achieving complication rates comparable to published data. These findings support the role of supervised augmentation mastopexy as a valuable component of plastic surgery training.

**Level of Evidence: 4 (Risk):**

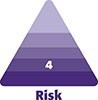

Hypomastia, defined as the underdevelopment of breast tissue, may result from congenital anomalies—such as primary hypoplasia, Poland syndrome, or chest wall deformities—or from acquired conditions, including postpartum involution, lactation-related atrophy, or significant weight loss.^1^ This condition affects a substantial number of women and may negatively influence body image, self-esteem, sexual satisfaction, and overall quality of life.

Silicone breast implants are widely used in the surgical management of hypomastia, providing reliable aesthetic and functional outcomes. Over time, surgical techniques have evolved to include combined procedures such as single-stage augmentation mastopexy. When appropriately indicated, performed in carefully selected patients, and executed by experienced surgeons, this combined approach has demonstrated safety and effectiveness comparable to staged procedures.^2^

Breast ptosis is defined as the descent of the breast parenchyma and the nipple–areola complex (NAC) below the inframammary fold, resulting in loss of youthful contour and upper pole fullness. Regnault classified breast ptosis into Grades I to III based on the position of the nipple relative to the inframammary fold and introduced the concept of pseudoptosis to describe cases in which the nipple remains at or above the fold, whereas the glandular tissue descends inferiorly.^3^ The degree of ptosis significantly influences both aesthetic presentation and surgical planning, often necessitating mastopexy or combined augmentation mastopexy to restore projection and correct tissue laxity.

Despite technical advancements, augmentation mammaplasty—particularly when combined with mastopexy—remains a technically demanding procedure and is associated with an inherent risk of complications, including hematoma, wound-healing disturbances, capsular contracture, implant malposition, and the need for reoperation.^4^ The increasing demand for aesthetic breast surgery has highlighted the importance of ensuring adequate training for plastic surgery residents.

Although resident involvement in reconstructive breast surgery is well established, participation in aesthetic procedures—especially those involving breast implants—remains more limited and controversial. Several studies have evaluated outcomes of breast surgery performed with resident participation, reporting mixed results. Data from large surgical registries, such as the American College of Surgeons National Surgical Quality Improvement Program, suggest that resident involvement, particularly during early stages of training, may be associated with a slight increase in major or wound-related complications in breast-reduction procedures.^5,6^ Conversely, other studies indicate that with structured supervision and progressive autonomy, residents can safely perform aesthetic breast procedures, achieving outcomes comparable to those of attending surgeons.^4^

Learning curve models, including that proposed by Carty et al, emphasize a progressive reduction in operative time, variability, and complication rates with increasing surgical experience, underscoring the importance of hands-on exposure as a fundamental component of surgical education.^7^ Furthermore, dedicated aesthetic surgery training programs—particularly in high-volume centers in Brazil—have demonstrated that residents can attain high levels of technical competence and procedural safety when directly involved in aesthetic procedures under structured guidance.^8^

The present study aims to analyze all cases of augmentation mastopexy performed over a 4-year period at the Hospital da Plástica in Rio de Janeiro, a reference center for aesthetic surgery training in Latin America. All procedures were performed by plastic surgery residents under the direct supervision of experienced faculty, using standardized techniques derived from the approaches of Pitanguy, Hakme, and Ribeiro.^9-11^

The primary objective is to evaluate the incidence and types of complications and to compare these findings with data reported in the literature. A secondary objective is to assess the educational implications of resident involvement, with particular attention to the role of trainees in implant-based breast surgery throughout the surgical learning curve.

## METHODS

### Study Population

Female patients undergoing augmentation mastopexy at the Hospital da Plástica in Rio de Janeiro, Brazil between January 2020 and January 2024 were considered eligible for inclusion. Inclusion criteria were age ≥18 years and a surgical indication for hypomastia, breast ptosis (at least partial areolar ptosis and/or NAC descent ≥2 cm), or aesthetic dissatisfaction of the breast, including cases related to postpartum involution or significant weight loss. American Society of Anesthesiologists (ASA) physical status classification was used to assess patients’ preoperative general health status, and patients with active smoking habits or relevant systemic comorbidities (ASA physical status >II) were excluded.

### Surgeon Population and Training Environment

All procedures included in the study were performed by senior plastic surgery residents (postgraduate year [PGY] ≥4) under direct supervision of attending surgeons with >20 years of experience in aesthetic and reconstructive plastic surgery, in accordance with institutional educational standards.

Preoperative consultations, including patient evaluation, counseling, and initial treatment proposals, were conducted by the residents. Each case was subsequently reviewed during a structured weekly surgical planning meeting attended by supervising faculty, during which indications, operative strategy, and implant selection were discussed and formally approved or modified as deemed appropriate by the attending surgeons. Final treatment decisions were validated by the supervising attending. During surgery, a supervising attending surgeon was physically present in the operating room for the entirety of the procedure. Postoperative visits, wound care, and follow-up assessments were primarily conducted by the residents, with attending involvement readily available and direct evaluation performed whenever clinical or patient concerns arose. All complications and revision decisions were discussed with and confirmed by the supervising attending surgeon. A standardized institutional perioperative protocol was applied to all procedures to ensure patient safety while preserving progressive surgical autonomy within a supervised training framework.

### Surgical Technique

The surgical approach was based on the classic Pitanguy mastopexy technique and its subsequent modifications, employing either superior or superomedial pedicles according to individual anatomical characteristics.^9^ Skin resection was performed using an inverted-T or reduced-pattern design, followed by deepithelialization of the marked area, excision of a central wedge of glandular tissue, and transposition of the NAC to Point A.

Reshaping of the remaining parenchyma was performed to enhance upper pole fullness and achieve stable breast projection. In selected cases, technical modifications described by Zavrides were adopted to improve flap mobility or enhance upper pole definition, particularly in patients with dense parenchyma or more severe ptosis.^10^ NAC transposition was performed using either a pure superior pedicle, as described by Letherman–Bostwick, or a superomedial pedicle according to Silveira, depending on anatomical considerations and the degree of transposition required, with the aim of optimizing NAC perfusion and aesthetic outcome.^12-14^

### Implant Characteristics and Placement

Implant selection was based on aesthetic goals, patient preferences, and soft-tissue characteristics. Preoperative evaluation, patient counseling, and preliminary implant sizing were performed by the resident surgeon. Each case was subsequently discussed collegially during a weekly departmental meeting with attending plastic surgeons, during which surgical indications, implant selection, and operative strategy were reviewed and confirmed. Final decisions regarding implant size, surface characteristics, and pocket placement were approved by the supervising attending surgeon. When needed, implant selection was further refined intraoperatively based on direct assessment of tissue characteristics, under continuous attending supervision.

Textured and polyurethane-coated implants were used. Implant insertion was performed through vertical, T-shaped, or L-shaped incisions, with placement in either a subglandular or a dual-plane pocket according to anatomical findings and the agreed surgical plan.

### Data Collection and Follow-Up

Postoperative follow-up visits were primarily conducted by the resident surgeon who had performed the procedure. Clinical data were collected retrospectively through structured outpatient follow-up visits, scheduled weekly during the immediate postoperative period and subsequently at progressively longer intervals. All patients were followed for a minimum of 1 year, allowing adequate assessment of both early and at least mid-term complications. Minor issues were managed by the resident within established institutional guidelines. In cases of diagnostic uncertainty, major complications, need for surgical revision, or patient concern, the case was directly discussed with and evaluated by the supervising attending surgeon. Decisions regarding reoperation were confirmed by the attending surgeon, who maintained overall responsibility for patient management.

All findings, including complications, reoperations, and aesthetic outcomes, were documented in the medical records by the surgical teams and supervising physicians. Data were manually extracted from clinical charts and operative reports and subsequently transcribed into a structured database.

### Ethical Considerations and Statistical Analysis

All patients provided written informed consent for surgery and for the anonymized use of their clinical data for research purposes, in accordance with the internal policies of the Hospital da Plástica. Ethical approval for the study was formally submitted and accepted through the Brazilian national research platform *Plataforma Brasil*. Patients who did not provide consent or failed to comply with the follow-up protocol were excluded. Descriptive statistical analysis was performed using Microsoft Excel (Microsoft Office 365, Microsoft Corporation, Redmond, WA).

## RESULTS

### Patient Population

Between January 2020 and January 2024, a total of 289 female patients underwent augmentation mastopexy and met inclusion criteria. All procedures were performed under general anesthesia. Patient age ranged from 22 to 64 years (mean age 43.4). No active smokers or patients with significant systemic comorbidities (ASA physical status >II) were included in the analysis. The mean follow-up duration was 18.3 months (range, 12-31 months).

### Surgeon Population

Overall, 26 plastic surgery trainees participated in the operative cases. All were licensed physicians fully integrated into the institution's daily clinical and educational activities. Most were Brazilian (84.6%), whereas the remaining trainees (15.4%) were from other countries.

Surgeon age ranged from 28 to 34 years. Continuous senior supervision was ensured by attending surgeons in accordance with institutional protocols. The main characteristics of the study design and methodology are summarized in Table 1. Key characteristics of the surgeon population are summarized in Table 2.

**Table 1.** Summary of Study Design and Methodology

Category	Details
Study period	January 2020–January 2024
Number of procedures	289 (augmentation mammaplasty or augmentation mastopexy)
Patient demographics	Female patients, age 22–64 years
Primary indication	Hypomastia, ptosis, and/or aesthetic dissatisfaction
Anesthesia	General anesthesia
Surgical technique	Classic Pitanguy technique^8^ with occasional Zavrides modifications^9^
Pedicle types	Superior pedicle (Letherman–Bostwick): 203 cases Superomedial pedicle (Silvera): 86 cases^10^

**Table 2.** Surgeon Population (Aggregated Data)

Variable	Value/description
Total number of trainees	26
Professional status	Licensed physicians
Nationality	Brazil (∼85%), other countries (∼15%)
Previous training	Brazilian: ≥2 years general surgery; International: ≥3 years surgical training including formal plastic surgery experience
Plastic surgery training level	2nd–3rd year (≥PGY 4 equivalent)
Age	28-34 years
Clinical involvement	Daily participation in clinical and educational activities
Senior supervision	Attending surgeons with >15 years' experience in aesthetic and reconstructive plastic surgery

### Surgical Technique and Implant Placement

NAC transposition was performed using 2 pedicle techniques: a pure superior pedicle in 203 cases, and a superomedial pedicle according in 86 cases. Pedicle selection was based on individual anatomy and the required degree of transposition, with the aim of optimizing NAC perfusion and aesthetic positioning.

Implant volumes ranged from 195 to 485 cc, with a mean volume of 307.2 cc. According to the ISO 14607:2018 implant surface classification, implant surfaces included microtextured and macrotextured devices. Specifically, 15 implants (5.2%) were macrotextured, 75 implants (26.0%) were macrotextured polyurethane coated (Silimed, Rio de Janeiro, Brazil), and 199 implants (68.9%) were microtextured (Silimed, Rio and Motiva, Establishment Labs, Alajuela, Costa Rica). Although polyurethane-coated implants are technically classified as macrotextured under ISO criteria, they were analyzed separately because of their distinct surface characteristics. Smooth implants were not used.

**Figure 1.**
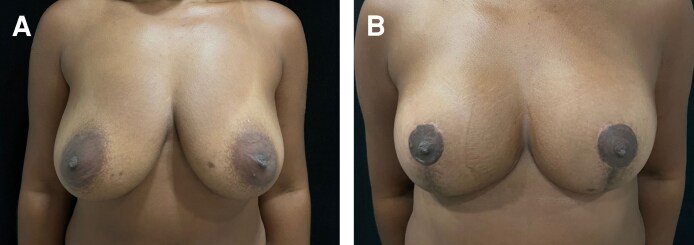
Representative outcome following subglandular augmentation mastopexy with 240 cc polyurethane implants in a 40-year-old female patient. (A) Preoperative view. (B) Postoperative view at 12 months follow-up demonstrating maintained projection, improved breast position, and scar maturation.

Access routes included 32 vertical or short-scar incisions and 257 T- or L-shaped incisions. Implant placement was subglandular in 88 cases (30.4%) and dual plane in 201 cases (69.6%), based on intraoperative assessment of soft-tissue quality. Figure 1 illustrates a representative postoperative result at 12 months following subglandular augmentation mastopexy performed within the structured training program.

### Complications and Reoperations

Mean operative time (skin to skin), excluding anesthesia-related procedures, was 204 min (range, 145-309 min). A total of 46 complications were recorded, corresponding to an overall complication rate of 15.9%. These included minor and major events as detailed in the following sections.

#### Minor Complications (*n* = 30; 10.4%)

Minor complications were calculated on a patient basis (*n* = 30). However, because some patients presented with >1 minor event (eg, concurrent seroma and wound dehiscence), the cumulative number of minor complication events exceeds the total number of affected patients.

Suture dehiscence was the most frequent complication, occurring in 14 cases (4.84%) and accounting for 30.4% of all complications. Clinical presentations ranged from limited wound separation to superficial NAC compromise. Dehiscence occurred in 4 cases with macrotextured polyurethane-coated implants placed in the subglandular plane (all >350 cc) and in 10 cases with microtextured implants (8 dual plane/partially submuscular and 2 subglandular).

**Figure 2.**
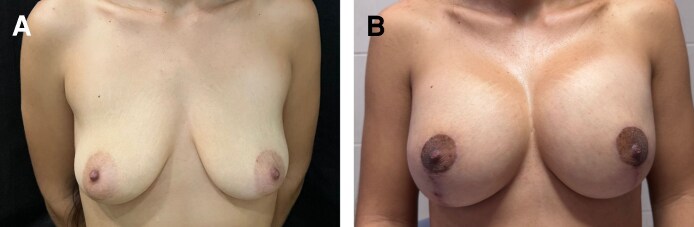
A 32-year-old female patient undergoing dual-plane mastopexy with 325 cc textured implants. (A) Preoperative front view. (B) Five months postoperative follow-up with mild secondary breast ptosis.

Secondary ptosis was reported in 10 cases (3.40%), equally distributed between macrotextured polyurethane-coated subglandular implants (215-435 cc) and microtextured dual-plane/submuscular implants (315-350 cc). In most cases, ptosis was mild and did not require surgical correction during follow-up (Figure 2).

**Figure 3.**
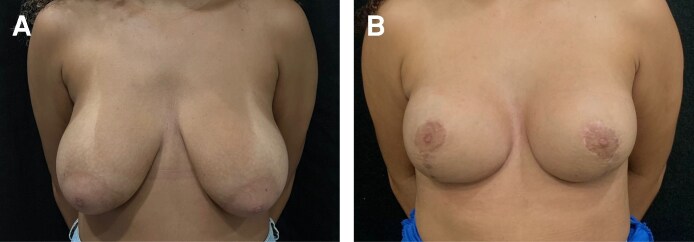
A 37-year-old female patient with breast volume loss and ptosis after 3 pregnancies. (A) Preoperative view. (B) Patient at 6 month follow-up. Mild nipple–areola complex ptosis with height asymmetry.

Asymmetry was observed in 9 cases (3.11%). NAC height discrepancy was noted in 6 cases (4 microtextured dual plane/submuscular, 1 polyurethane subglandular, and 1 textured dual plane/submuscular), whereas lateralization or rotation occurred in 3 cases (all with microtextured dual-plane/submuscular implants; Figure 3). Seroma developed in 4 cases (1.30%) and was managed conservatively in all patients; in 3 cases, seroma was associated with concurrent suture dehiscence.

#### Major Complications (*n* = 16; 5.5%)

Hematoma occurred in 4 patients (1.30%), representing 8.6% of all complications. All cases involved implants >325 cc and required surgical evacuation: 3 cases involved microtextured dual-plane/submuscular implants and 1 a polyurethane-coated subglandular implant.

**Figure 4.**
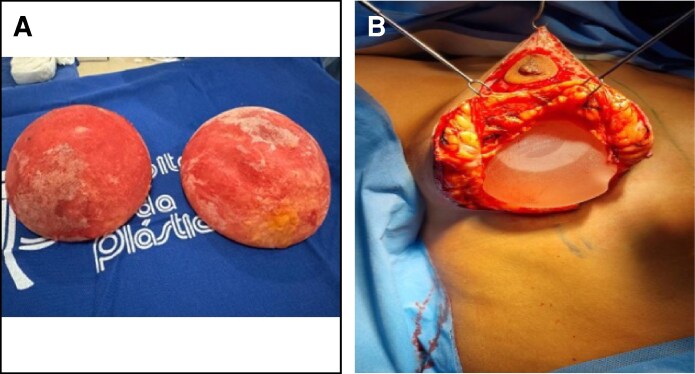
Intraoperative images of Baker grade III capsular contracture in a 46-year-old female patient. (A) Implant removal. (B) Revision mastopexy with implant exchange.

Capsular contracture (Baker grade III) was diagnosed in 2 cases (0.70%). One case involved a 260 cc microtextured dual-plane/submuscular implant placed in 2020 and diagnosed in 2022; the other involved a 305 cc macrotextured polyurethane-coated subglandular implant developing contracture at 1 year (Figure 4). Both patients underwent revision surgery.

**Figure 5.**
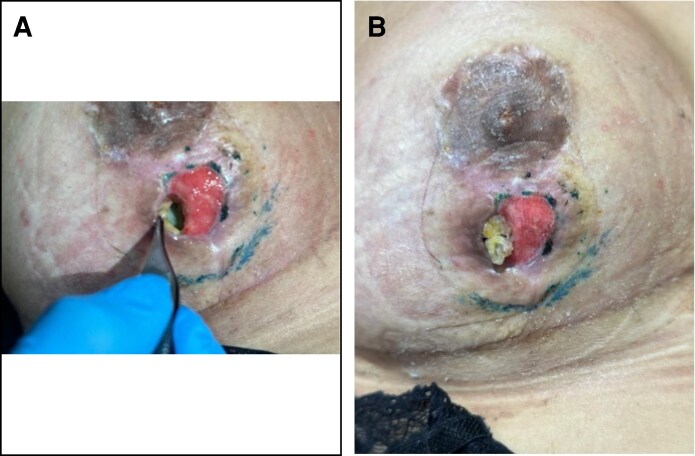
A 51-year-old female patient at 40-day postoperative follow-up. Wound dehiscence complicated by infection and implant exposure. (A) Physician indicating the undermined wound with forceps. (B) Low frontal view of the wound dehiscence showing fibrin deposition and granulation tissue.

Infection occurred in 3 cases (1.00%), accounting for 6.5% of all complications. In 2 cases, implant exposure secondary to wound dehiscence required implant removal. Both cases involved macrotextured polyurethane-coated subglandular implants (305 and 330 cc; Figure 5).

**Figure 6.**
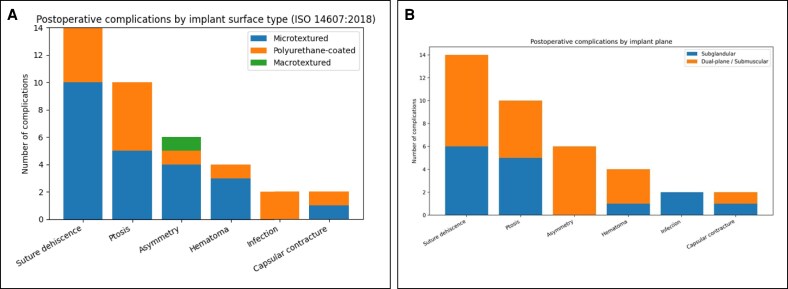
Distribution of postoperative complications by implant surface type and implant plane. (A) Complications stratified according to implant surface classification following ISO 14607:2018, including microtextured, macrotextured, and macrotextured polyurethane-coated implants. (B) Complications stratified according to implant plane (subglandular vs dual plane/submuscular). Absolute numbers of complications are shown. ISO: International Organization for Standardization.

A total of 11 reoperations (3.8%) were performed, including all major complications and selected minor complications not resolved with conservative treatment or at patient request because of aesthetic dissatisfaction. All revision procedures were performed at the same institution with attending surgeon involvement. Details of complications and subsequent interventions are summarized in Table 3 and Figure 6.

**Table 3.** Summary of Complications

Complication	No. of cases (*n*)	Percentage	Classification	Management
Suture dehiscence	14	4.84	Minor	Conservative (dressing changes, some minor necrosis; no reoperation)
Ptosis	10	3.40	Minor	Observed; no surgical correction during follow-up
Asymmetry	9	3.11	Minor	Conservative; occasional revision on demand
Seroma	4	1.30	Minor	Managed conservatively; aspiration if needed
Hematoma	4	1.30	Major	Required surgical evacuation
Capsular contracture	2	0.70	Major	Reoperation required
Infection	3	1.00	Major	Two cases with implant exposure → explantation

## DISCUSSION

### Interpretation of Findings and Comparison With the Literature

Augmentation mammaplasty, particularly when combined with mastopexy, remains a technically demanding procedure associated with a meaningful risk of postoperative complications. In this series of 289 single-stage augmentation mastopexies performed over 4 years in a teaching environment, the overall complication rate was 15.9%, which is broadly consistent with published data. Khavanin et al reported, in a systematic review including 4856 single-stage cases, an overall complication rate of 13.1% and a reoperation rate of 10.65%, with recurrent ptosis (5.2%), capsular contracture (2.97%), asymmetry (2.94%), seroma (1.42%), hematoma (1.37%), and infection (0.93%) as the most commonly reported events.^15^ Our complication profile was comparable, although the overall rate was slightly higher, which may reflect the fact that all procedures were performed by trainees under supervision.

Suture dehiscence was the most frequent complication (4.84%), exceeding the 1% to 2% rate reported in large retrospective analyses.^15-17^ This finding likely reflects the combined influence of high-tension closures at the T-junction, implant size, implant surface characteristics (polyurethane-coated macrotextured or microtextured), and the intrinsic vulnerability of mastopexy incision patterns. In line with this, Stevens et al reported that T-junction breakdown is more common in augmentation mastopexy cases involving more extensive skin resections.^16^

Secondary ptosis occurred in 3.4% of cases, comparable to the pooled recurrence rate reported by Khavanin et al (5.2%).^15^ Ptosis is more likely in patients with larger or heavier implants, reduced dermal quality, and insufficient internal support. In our cohort, ptosis was observed mainly in patients receiving macrotextured or microtextured implants, both in subglandular and dual-plane/submuscular positions, particularly when implant volume exceeded 350 cc.

The incidence of relapsing ptosis observed in the present cohort was lower than that reported in most published series, despite procedures being performed by trainees under direct supervision. This finding should be interpreted in light of the definition adopted in our study. Relapsing ptosis was recorded based on clinical assessment during follow-up and did not necessarily imply the need for surgical revision. Reports in the literature define recurrent ptosis primarily in the context of reoperation rates or patient-driven revision requests, which may partially account for the higher incidences described.

Furthermore, the minimum follow-up of 12 months, while sufficient to detect early and intermediate recurrences, may underestimate late-onset ptosis, which has been reported to occur up to several years after augmentation mastopexy. This temporal limitation has been acknowledged and discussed as an inherent constraint of the study design. Notably, the similar distribution of relapsing ptosis between subglandular and dual-plane implant placement suggests that this complication may be more strongly influenced by tissue quality, baseline ptosis severity, and soft-tissue biomechanics rather than by the implant plane itself. Within these limits, the low incidence observed in our series may reflect a standardized surgical approach and structured supervision in a training environment, rather than a true deviation from expected long-term outcomes.

Asymmetry (3.11%) remains a frequent cause of aesthetic dissatisfaction. Differences in NAC height or implant position may become more apparent during postoperative settling and are often multifactorial, including baseline asymmetry and soft-tissue behavior. Qureshi et al emphasized that minor asymmetry is nearly universal and may be accentuated by surgery.^18^ In our experience, most asymmetries were managed conservatively, with selected revisions performed at patient request.

Hematoma occurred in 1.3% of patients, consistent with the 1% to 2% range reported in the literature.^16,19^ Most cases involved implant volumes ≥325 cc. Larger implants and subglandular placement have been associated with increased bleeding risk.^15^ All hematomas were identified promptly and managed with surgical evacuation.

Seroma was observed in 1.3% of cases and managed conservatively. Published rates range from 3% to 6%, particularly with textured or polyurethane-coated implants.^16,20^ Our lower incidence may be related to meticulous hemostasis and dead-space control, potentially facilitated by dual-plane pocket creation.

Capsular contracture was diagnosed in only 2 patients (0.7%), both Baker grade III, which is below the pooled rate reported in meta-analyses (2.97%).^15,21^ Both cases required revision surgery. Standard preventive measures were consistently applied, including glove change, pocket irrigation, and atraumatic implant handling. Although the literature suggests that submuscular placement and specific implant surfaces may reduce the risk of contracture, contracture remains multifactorial and cannot be fully eliminated.^15-17^

Infection occurred in 1.0% of cases, within the commonly reported range of 1% to 3%.^16,22^ Two infections required explantation because of implant exposure following wound dehiscence. These findings reinforce the importance of meticulous aseptic technique and standardized perioperative protocols, particularly in procedures involving trainees. The literature most frequently implicates *Staphylococcus aureus* and *Staphylococcus epidermidis* in implant-related infections, consistent with perioperative contamination as a key mechanism.^17,22^

Notably, our reoperation rate was 3.8%, substantially lower than the range of 10% to 16% reported in several series.^15-17^ Reoperations were primarily performed for major complications (hematoma, infection with exposure, and capsular contracture) and for selected minor complications driven by patient dissatisfaction. A lower revision rate may reflect effective conservative management, structured follow-up, and careful perioperative counseling with expectation management.

Although implant volume was not analyzed as a formal stratification variable, several major complications in our series occurred in patients receiving larger implants. Future studies with adequate power and predefined volume thresholds are warranted to better define the relationship between implant size and complication risk, particularly in a training environment.

A comparison between our complication rates and those reported in the literature is summarized in Table 4.

**Table 4.** Comparison of Complication Rates With Published Literature

Complication	Present study (%)	Literature (%)	Comment
Overall complication rate	15.9	Foustanos et al^14^ (13.1)	Slightly higher; may reflect trainee-performed cases under supervision and/or broader capture of minor events
Suture dehiscence	4.84	Foustanos et al,^14^ Khavanin et al,^15^ Stevens et al^16^ (1-2)	Higher than most reports; likely influenced by T-junction tension, implant volume, and soft-tissue characteristics
Secondary ptosis	3.4	Foustanos et al^14^ (5.2)	Comparable; typically associated with larger implants and reduced soft-tissue support
Asymmetry	3.11	Foustanos et al,^14^ Calobrace et al^17^ (2.9-5.0)	Within expected range; often related to baseline asymmetry and postoperative tissue dynamics
Hematoma	1.3	Foustanos et al,^14^ Khavanin et al,^15^ Qureshi et al^18^ (1-2)	Consistent with literature; all cases required surgical evacuation in this series
Seroma	1.3	Khavanin et al,^15^ Hall-Findlay^19^ (3-6)	Lower than commonly reported; may relate to meticulous hemostasis and dead-space control (eg, dual-plane pocket)
Infection	1.0	Khavanin et al,^15^ Hall-Findlay^21^ (1-3)	Consistent with literature; 2 cases required explantation because of exposure

### Safety of Trainee Involvement: Evidence From Other Surgical Specialties

Our findings are consistent with broader surgical literature evaluating trainee participation across specialties. Multiple studies have shown that resident or fellow involvement may increase operative time yet does not necessarily translate into higher major complication rates when direct supervision and standardized protocols are in place.

In orthopedics, a large retrospective analysis of 1743 revision total hip arthroplasties found that trainee participation was associated with longer operative time but not with increased acute postoperative complications.^23^ Similarly, in pediatric otolaryngology, analyses including >7600 tonsillectomies reported longer operative times for trainees but no significant differences in readmissions or postoperative hemorrhage, including cases performed by more junior residents; operative time decreased progressively with advancing training level (PGY1 to PGY4) without an increase in complications.^24^ In gynecology, trainee participation in vaginally assisted laparoscopic procedures has likewise been reported as not increasing intra- or postoperative complications despite longer procedures.^25^ Comparable findings have been reported in elective high-complexity orthopedic procedures such as primary total hip arthroplasty, where outcomes were not adversely affected and operative efficiency improved with experience.^26^

Collectively, these data support a central concept: the safety of trainee participation is primarily determined by the quality of supervision, institutional protocols, and structured progression of autonomy, rather than by procedure complexity alone. Applied to the present cohort, the observation that complication rates were comparable to—or in some domains lower than—those reported in series performed exclusively by attending surgeons supports the feasibility of safely performing single-stage augmentation mastopexy in a structured teaching setting, provided continuous supervision and standardized perioperative pathways are ensured.

### Study Limitations

This study has limitations inherent to its retrospective design. First, reliance on clinical documentation may lead to underreporting of minor or transient complications. Second, the cohort reflects the experience of a single institution in which all procedures were performed by trainees under direct supervision; therefore, outcomes may not be generalizable to settings with different training structures or supervision intensity. Third, potential confounders such as BMI, previous smoking history, and comorbidities were not stratified in this preliminary analysis.

An additional limitation of this study is the duration of follow-up. Only patients with a minimum follow-up of 12 months were included, allowing reliable detection of early- and mid-term complications. However, late complications such as implant malposition, recurrent ptosis, or capsular contracture may develop beyond this time frame. Longer follow-up studies are therefore warranted to fully assess long-term outcomes.

Implant volume was not included as a primary stratification variable; therefore, volume-related risk assessment should be interpreted cautiously and represents an area for future investigation.

Importantly, all procedures were performed within a structured academic program with direct attending supervision. Previous studies have shown that resident participation in surgical procedures does not significantly increase major complication rates,^24-26^ particularly within structured surgical planning protocols^27^ and under direct attending surgeon supervision.^27^

The degree of attending surgeon involvement likely contributed to the observed outcomes and should be considered when interpreting the results. The present study reflects a model of progressive autonomy within a structured supervision framework rather than unsupervised resident practice. Within this model, retrospective complication analysis represents a valuable educational and quality-improvement tool: systematic monitoring allows training centers to benchmark outcomes, identify patterns of risk, refine technical and perioperative protocols, and reinforce patient safety as a core principle of surgical education. Wider adoption of structured, data-driven performance review, particularly when compared with literature, may help academic centers promote transparency, continuous improvement, and surgical excellence.

## CONCLUSIONS

In this retrospective series of 289 augmentation-mastopexy procedures performed in a structured teaching environment, complication rates remained within acceptable limits and were broadly comparable to published data. These findings support the effectiveness of direct attending supervision, standardized operative protocols, and progressive, controlled hands-on training in enabling residents to safely perform augmentation-mastopexy procedures.

Beyond clinical outcomes, this study highlights the value of structured complication analysis as an instrument for institutional self-assessment and continuous quality improvement in surgical education. Objective performance monitoring may help training programs identify risk patterns, refine educational strategies, and promote a culture of accountability and excellence in resident training.
